# Human Lacrimal Gland Gene Expression

**DOI:** 10.1371/journal.pone.0169346

**Published:** 2017-01-12

**Authors:** Vinay Kumar Aakalu, Sowmya Parameswaran, Mark Maienschein-Cline, Neil Bahroos, Dhara Shah, Marwan Ali, Subramanian Krishnakumar

**Affiliations:** 1 Lacrimal Cell Biology Laboratory, University of Illinois at Chicago, Department of Ophthalmology and Visual Sciences, Chicago, Illinois, United States of America; 2 Radheshyam Kanoi Stem Cell Laboratory, Vision Research Foundation, Kamalnayan Bajaj Institute for Research in Vision and Ophthalmology, Chennai, Tamil Nadu, India; 3 Research Informatics Core, Research Resources Center, University of Illinois at Chicago, Chicago, Illinois, United States of America; Wayne State University School of Medicine, UNITED STATES

## Abstract

**Background:**

The study of human lacrimal gland biology and development is limited. Lacrimal gland tissue is damaged or poorly functional in a number of disease states including dry eye disease. Development of cell based therapies for lacrimal gland diseases requires a better understanding of the gene expression and signaling pathways in lacrimal gland. Differential gene expression analysis between lacrimal gland and other embryologically similar tissues may be helpful in furthering our understanding of lacrimal gland development.

**Methods:**

We performed global gene expression analysis of human lacrimal gland tissue using Affymetrix ^®^ gene expression arrays. Primary data from our laboratory was compared with datasets available in the NLM GEO database for other surface ectodermal tissues including salivary gland, skin, conjunctiva and corneal epithelium.

**Results:**

The analysis revealed statistically significant difference in the gene expression of lacrimal gland tissue compared to other ectodermal tissues. The lacrimal gland specific, cell surface secretory protein encoding genes and critical signaling pathways which distinguish lacrimal gland from other ectodermal tissues are described.

**Conclusions:**

Differential gene expression in human lacrimal gland compared with other ectodermal tissue types revealed interesting patterns which may serve as the basis for future studies in directed differentiation among other areas.

## Introduction

The lacrimal gland and its secretory acini are critical to the production of tears and in particular the aqueous layer of the tear film [1,2]. Although studies on human lacrimal gland biology have been undertaken for many years, progress has been slow due to difficulties in obtaining tissue samples, small and often diseased tissue samples and limited availability of tissue and cell type specific markers for lacrimal gland cell types. In particular, the limited available data on gene expression in the human lacrimal gland has driven a number of studies investigating the expression of genes in specific diseases which affect the lacrimal gland and in search of markers for lacrimal epithelium [3–4]. Understanding of the development of the lacrimal gland is important, but limited. Various studies have been undertaken to evaluate transcription factors involved in murine lacrimal gland development and in fetal morphogenic changes in human lacrimal gland, but further study in gene expression involved in the development of human lacrimal gland is necessary [5–6].

Studies in other exocrine tissues such as salivary gland have demonstrated the transformative potential of regenerative therapies in previously recalcitrant diseases. Strategies have ranged from the use of collected slow-cycling precursors to the utilization of embryonic stem cell and induced pluripotent stem cell (iPSC) technologies [7]. In particular, development of directed differentiation protocols in other ectodermal tissues has shown increasing promise for the development of cell based therapies [8].

iPSC based therapies are often driven by an understanding of the critical factors involved in the development of specialized cell and tissue types. Differentiation along pathways that mimic the normal development of these tissues is undertaken through the use of various methods, including exogenous factors. Success in this arena has led to the development of disease models and drug screening as well as therapeutic measures [9].

Studies have begun in earnest with the goal of developing regenerative and cell based treatments fordry eye disease and some have focused on lacrimal gland regeneration [2]. Although progress has been made, the development of human lacrimal gland regenerative technologies has lagged behind developments in animals [10–11]. Moreover, the lack of known lacrimal epithelial biomarkers and critical factors in human lacrimal gland development hamper progress in the development of potential cell and stem cell based therapies.

A better understanding of human lacrimal gene expression can drive future studies on lacrimal gland development, development of therapeutic targets, and drug development. In this study we sought to develop a better understanding of lacrimal gland gene expression as it compares with developmentally related, surface ectoderm derived tissues including cornea, conjunctiva, epidermis and salivary gland tissues.

## Methods

### Ethics Statement

For accessory lacrimal gland tissue utilized in this study, written informed consent was obtained from patients using a consent form specifically approved for this study by the Institutional Review Board (IRB) and processed by The University of Illinois at Chicago (UIC). Completed, signed consent forms were maintained according to the university guidelines following an IRB approved protocol specific for this study. Cadaver donor main lacrimal glands, included in this study were de-identified, and provided anonymously by the National Disease Research Interchange (ndriresource.org) and use of these tissues was sanctioned by the UIC IRB. The protocol number for the approved IRB # 2009–0832. The remainder of the data sets were obtained anonymously through the NCBI Gene Expression Omnibus (https://www.ncbi.nlm.nih.gov/geo/).

### Description of Sample Preparation

Main lacrimal glands from three cadaveric donors (5 total glands) were prepared for RNA extraction. Accessory lacrimal glands from one patient preserved in formalin fixed paraffin embedded tissue (FFPE) were obtained using LASER Capture Microdissection as described by our laboratory previously [7]. Biological duplicates are noted. Table 1 describes the details of the samples utilized in this study.

**Table 1:** Tissue Samples and Data Sets.

GEO Study ID	GEO Dataset ID	Tissue Type	Array Platform	Details
**GSE89827**	GSM2390107	Main Lacrimal Gland	HTA2.0	Right Side
**GSE89827**	GSM2390108	Main Lacrimal Gland	HTA2.0	Left Side
**GSE89827**	GSM2390109	Main Lacrimal Gland	HTA2.0	Right Side
**GSE89827**	GSM2390110	Main Lacrimal Gland	HTA2.0	Left Side
**GSE89827**	GSM2390113	Main Lacrimal Gland	HTA2.0	
**GSE89827**	GSM2390114	Main Lacrimal Gland	HTA2.0	Biologic Duplicate
**GSE89827**	GSM2390111	Accessory Lacrimal Gland	HuGene 1.0	FFPE
**GSE89827**	GSM2390112	Accessory Lacrimal Gland	HuGene 1.0	FFPE Biologic Duplicate
**GSE58331**	GSM1407233	Main Lacrimal Gland	pd.hg.u133.plus.2	Lacrimal gland 15_15ANo
**GSE58331**	GSM1407235	Main Lacrimal Gland	pd.hg.u133.plus.2	Lacrimal gland 17_17ANo
**GSE58331**	GSM1407236	Main Lacrimal Gland	pd.hg.u133.plus.2	Lacrimal gland 18_18ANo
**GSE58331**	GSM1407237	Main Lacrimal Gland	pd.hg.u133.plus.2	Lacrimal gland 19_19ANo
**GSE58331**	GSM1407238	Main Lacrimal Gland	pd.hg.u133.plus.2	Lacrimal gland 20_20ANo
**GSE58331**	GSM1407240	Main Lacrimal Gland	pd.hg.u133.plus.2	Lacrimal gland 22_22ANo
**GSE58331**	GSM1407243	Main Lacrimal Gland	pd.hg.u133.plus.2	Lacrimal gland 25_25ANo
**GSE40568**	GSM1389618	Salivary	pd.hg.u133.plus.2	LSG excised from healthy volunteer, clinical sample1
**GSE40568**	GSM1389619	Salivary	pd.hg.u133.plus.2	LSG excised from healthy volunteer, clinical sample2
**GSE40568**	GSM1389620	Salivary	pd.hg.u133.plus.2	LSG excised from healthy volunteer, clinical sample3
**GSE23117**	GSM569473	Salivary	pd.hg.u133.plus.2	non-SS control gland, patient 16, batch 2
**GSE23117**	GSM569474	Salivary	pd.hg.u133.plus.2	non-SS control gland, patient 17, batch 2
**GSE23117**	GSM569475	Salivary	pd.hg.u133.plus.2	non-SS control gland, patient 18, batch 2
**GSE23117**	GSM569476	Salivary	pd.hg.u133.plus.2	non-SS control gland, patient 19, batch 2
**GSE36280**	GSM902385	Salivary	pd.hg.u133.plus.2	
**GSE36280**	GSM902386	Salivary	pd.hg.u133.plus.2	
**GSE36280**	GSM902387	Salivary	pd.hg.u133.plus.2	
**GSE40611**	GSM997850	Salivary	pd.hg.u133.plus.2	
**GSE40611**	GSM997851	Salivary	pd.hg.u133.plus.2	
**GSE40611**	GSM997852	Salivary	pd.hg.u133.plus.2	
**GSE40611**	GSM997853	Salivary	pd.hg.u133.plus.2	
**GSE40611**	GSM997854	Salivary	pd.hg.u133.plus.2	
**GSE40611**	GSM997855	Salivary	pd.hg.u133.plus.2	
**GSE40611**	GSM997856	Salivary	pd.hg.u133.plus.2	
**GSE40611**	GSM997857	Salivary	pd.hg.u133.plus.2	
**GSE40611**	GSM997858	Salivary	pd.hg.u133.plus.2	
**GSE40611**	GSM997859	Salivary	pd.hg.u133.plus.2	
**GSE40611**	GSM997860	Salivary	pd.hg.u133.plus.2	
**GSE40611**	GSM997861	Salivary	pd.hg.u133.plus.2	
**GSE40611**	GSM997862	Salivary	pd.hg.u133.plus.2	
**GSE40611**	GSM997863	Salivary	pd.hg.u133.plus.2	
**GSE40611**	GSM997864	Salivary	pd.hg.u133.plus.2	
**GSE40611**	GSM997865	Salivary	pd.hg.u133.plus.2	
**GSE40611**	GSM997866	Salivary	pd.hg.u133.plus.2	
**GSE40611**	GSM997867	Salivary	pd.hg.u133.plus.2	
**GSE71320**	GSM1832613	Cornea Epithelium	pd.hg.u133.plus.2	HCLE_LB control 1
**GSE71320**	GSM1832614	Cornea Epithelium	pd.hg.u133.plus.2	HCLE_LB control 2
**GSE29402**	GSM724096	Cornea Epithelium	pd.hg.u133.plus.2	Human Cornea Donor 1
**GSE29402**	GSM724097	Cornea Epithelium	pd.hg.u133.plus.2	Human Cornea Donor 2
**GSE29402**	GSM724098	Cornea Epithelium	pd.hg.u133.plus.2	Human Cornea Donor 15 OS (Left Eye)
**GSE29402**	GSM724093	Conjunctiva	pd.hg.u133.plus.2	Human Conjunctiva Donor 1
**GSE29402**	GSM724094	Conjunctiva	pd.hg.u133.plus.2	Human Conjunctiva Donor 2
**GSE29402**	GSM724095	Conjunctiva	pd.hg.u133.plus.2	Human Conjunctiva Donor 15 OS (Left Eye)
**GSE53751**	GSM1299978	human keratinocytes	pd.hg.u133.plus.2	Human primary keratinocytes, vehicle, bio rep 1
**GSE53752**	GSM1299979	human keratinocytes	pd.hg.u133.plus.2	Human primary keratinocytes, vehicle, bio rep 2
**GSE66359**	GSM1620805	human keratinocytes	pd.hg.u133.plus.2	Normal human epidermal keratinocyte 42 (cell line)
**GSE66359**	GSM1620806	human keratinocytes	pd.hg.u133.plus.2	Normal human epidermal keratinocyte 51 (cell line)
**GSE66359**	GSM1620807	human keratinocytes	pd.hg.u133.plus.2	Normal human epidermal keratinocyte 59 (cell line)
**GSE66359**	GSM1620808	human keratinocytes	pd.hg.u133.plus.2	Normal human epidermal keratinocyte 65 (cell line)
**GSE66359**	GSM1620809	human keratinocytes	pd.hg.u133.plus.2	NEHKPC (cell line) untreated
**GSE76446**	GSM2024775	human keratinocytes	pd.hg.u133.plus.2	Human primary keratinocytes, vehicle, bio rep 1
**GSE76446**	GSM2024776	human keratinocytes	pd.hg.u133.plus.2	Human primary keratinocytes, vehicle, bio rep 2
**GSE36287**	GSM886433	human keratinocytes	pd.hg.u133.plus.2	Keratinocytes Untreated Replicate 1
**GSE36287**	GSM886434	human keratinocytes	pd.hg.u133.plus.2	Keratinocytes Untreated Replicate 2
**GSE36287**	GSM886435	human keratinocytes	pd.hg.u133.plus.2	Keratinocytes Untreated Replicate 3

“Main lacrimal glands were obtained from cadaveric donors through the National Disease Research Interchange. All tissues were obtained within 18 hours of death of the donor and transmitted directly to the Lacrimal Cell Biology Laboratory on wet ice. Upon arrival, a fellowship trained Oculoplastic Surgeon (VKA) inspected the samples and, under microscopic guidance, dissected glandular tissue from the minimal surrounding stromal and adnexal tissues.”

For non-FFPE samples, Total RNA was extracted from human lacrimal gland tissue using RNeasy from QIAGEN using the manufacturer’s protocol. Extracted RNA was analyzed using Qubit quantification and Tape Station 2200 quantification-sizing quality control system. A cDNA library was created and labelled. Labeled double stranded cDNA was hybridized and analyzed on “Human Transcriptome Array (HTA) 2.0” Array from Affymetrix ^®^.

For FFPE samples, following RNA extraction, 25ng of total RNA was amplified, converted to cDNA, and labelled with “SensationPlus^™^ FFPE Amplification and WT Labeling Kit” from Affymetrix ^®^. The labeled double stranded cDNA was hybridized and analyzed on “GeneChip1 Human Gene (HuGene) 1.0 ST” Array from Affymetrix ^®^.

### Dataset Search

We searched the NLM GEO database for human gene expression data sets using Affymetrix ^®^ microarrays for each tissue type. Multiple laboratories were selected when possible for each tissue type and no disease phenotype samples were chosen. Skin samples consisted of epidermal keratinocytes, salivary glandular tissue consisted of whole glandular tissue, corneal samples consisted of corneal epithelium. Conjunctival samples included conjunctival epithelium and the immediately adjacent stroma. All samples utilized in this study were from non-diseased patients. These data sets were utilized as comparisons to samples prepared in our laboratory and further details are noted in Table 1.

### Raw Data Normalization and Annotation

Because the Affymetrix ^®^ arrays obtained for analysis consisted of a number of different array versions, we performed a multi-step normalization and cross-annotation procedure to obtain comparable expression levels. First, we obtained transformed raw RMAs using the R oligo package for each array individually: arrays were duplicated and expression levels were obtained using the rma function for each duplicate set of arrays (the rma function requires at least 2 arrays to be provided). The probe sets in the expression table for each array were annotated with array-type-specific probe set annotation csv files obtained from Affymetrix ^®^’s website, and the duplicated expression entries were removed. The various microarray datasets in this study ranged over 4 array types: pd.hg.u133a.2, pd.hg.u133.plus.2, pd.hta.2.0, and pd.hugene.1.0. To match gene annotations across array types, we compared the “Gene Symbol” columns in pd.hg.u133a.2 and pd.hg.u133.plus.2 with the “gene_assignment” columns in pd.hta.2.0 and pd.hugene.1.0. Any probe sets pairs between platforms that shared at least one gene name in these columns was included, and only the first gene assignment to a probe set was used within a given microarray. After merging these annotations, 12,398 genes were retained that could be cross-compared between platforms, for 112 samples (104 publicly available, plus 8 from this study). We then performed quantile normalization on the merged gene expression table using internal scripts: a reference distribution of expression levels was obtained across all 112 samples, and the quantiles for each individual sample were then matched to this reference distribution.

### Statistical Analysis

For this study, we focused on tissues that share a similar developmental context with lacrimal gland: salivary gland, cornea epithelium, conjunctiva, and skin. For each of these tissues, we performed a pair-wise comparison of expression levels with the lacrimal gland samples using both a t-test and a Mann-Whitney U test. After correcting for multiple testing using the false discovery rate (FDR) correction, we considered genes to be *differentially expressed* in lacrimal gland tissue relative to another tissue if the FDR was less than 0.01 for both the t-test and the U test. We further considered genes to be *lacrimal gland-specific* if they were differently expressed (FDR<0.01 for t-test and U test) for all four tissue comparisons. We also computed fold-changes between average expression levels in lacrimal gland versus each of the other four tissues, and considered genes to be up- or down-regulated in lacrimal gland if the fold changes were consistently higher or lower in lacrimal gland, respectively, across tissues. The top genes were obtained by ranking genes based on the average fold-change (averaged across each of the four tissue comparisons), and taking the most strongly up- and down-regulated genes.

### Pathway Analysis and Drug Targets

Cell surface markers were obtained by comparing the lacrimal gland-specific genes to the “cell surface” (GO:0009986) ontology in the Gene Ontology Cellular Component database [12]. Additional systems biology analysis was conducted using MetaCore^®^ [13]. Enrichment of canonical biological pathways among the lacrimal gland-specific genes was obtained using the “Pathway Maps” database in MetaCore^®^. We also looked for potential druggable targets within the lacrimal gland-specific genes: genes encoding for key regulatory molecules were identified from the MetaCore^®^ molecular annotations for transcription factor, regulators (GDI, GAP, GEF), GPCR, generic binding protein, generic enzyme, generic kinase, generic phosphatase, generic protease, or generic receptor. We then obtained drug targets for the resulting list of 323 genes in MetaCore^®^ [12–15].

For identifying the genes encoding secretory proteins, the differentially expressed gene list was analyzed using Human Protein Reference database [16]. The subcellular localization of each protein was obtained. The list of genes encoding for secreted protein with extracellular localization were analyzed for their expression status in the different tissues and represented in the form of heat-map [17].

## Results

**Fig 1:**
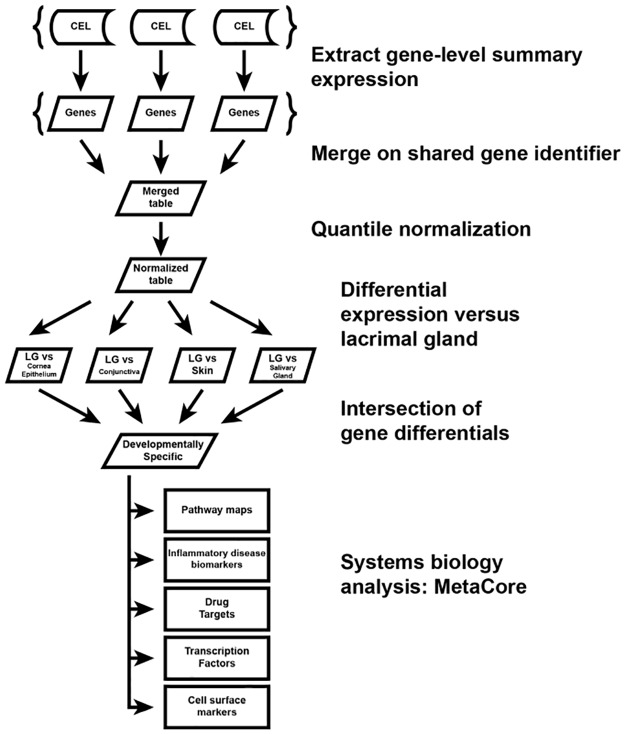
Schematic Illustration of Experimental Work-Flow.

Table 1 lists the samples, sources and tissue types of human tissue used for gene expression analysis and comparison. The work flow for analysis is described in Fig 1 and in the Methods. Briefly, the normalized gene expression data for each tissue type was compared with one another. Pair-wise comparisons were analyzed between each tissue type and lacrimal gland, and genes that were differentially expressed in all pair-wise comparisons (i.e. differentially expressed when comparing lacrimal gland to each specific tissue type) were genes were further analyzed for establishing lachrymal tissue-specific markers and signaling pathways involved in its development.

**Fig 2:**
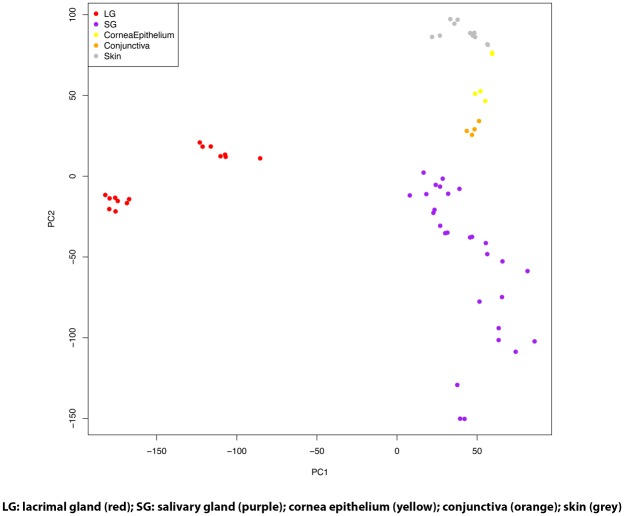
Principal Component Analysis of Tissue Transcriptomes.

After normalization of data, orthogonal transformation of data was performed and demonstrated in principal component analysis (PCA) in Fig 2. Despite the diversity of microarray versions and studies present in the data, the samples strongly cluster by tissue type, indicating that normalization procedures used were appropriate for obtaining quantitatively comparable gene expression estimates. In addition, the accessory lacrimal gland sample clustered well with the main lacrimal gland samples from our laboratory indicating limited differentiation of gene expression between these tissue types in this study. Notable is the significant difference noted between lacrimal gland tissue gene expression and all other tissue types. This may indicate that lacrimal gland gene expression is significantly different from the other tissue types noted.

**Fig 3:**
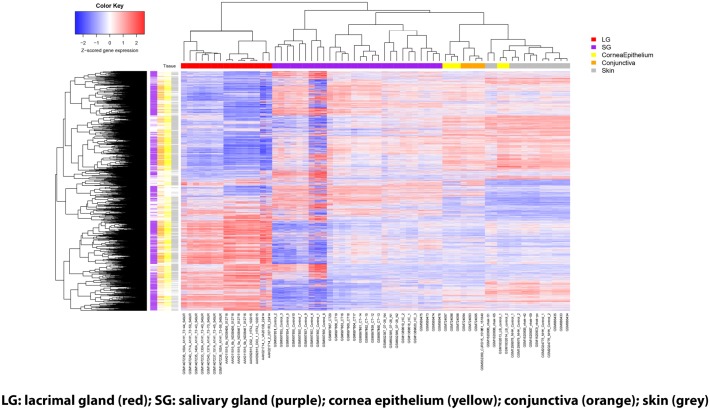
Heat-Map of Gene Expression Analysis of Lacrimal Gland and Other Ectodermal Tissues. Heat-Map showing genes with significant differential expression (FDR<0.01 for both t-test and U test) for all genes studied. The heat-map shows all the genes that were significant in at least one of the comparisons, which was 7787 total. The number significant in the LG vs all other comparisons (LG-specific genes) was 1855. The tissue types are color coded: bars along the top indicate which tissue each sample belongs to; bars down the left-hand side indicate which tissues were significantly differentially expressed with respect to LG for each gene.

Once demonstration that lacrimal gland does indeed have significantly different gene expression patterns from the comparison tissues, we sought to define the magnitude and types of differential gene expression. Fig 3 is a heat-map of gene expression across all tissues, showing only the genes that were differentially expressed in lacrimal gland with respect to at least one other tissue; colored bars on the left-hand side indicate when a gene was differentially expressed for each tissue comparison.

**Fig 4:**
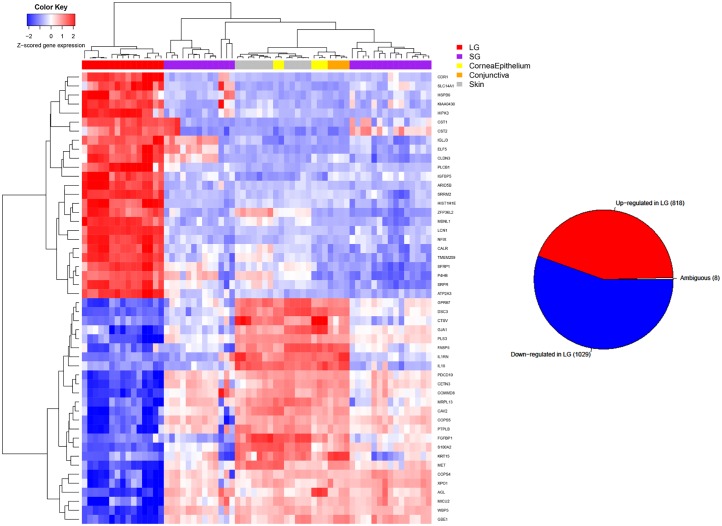
Heat Map of Top-50 Genes Expressed in Lacrimal Gland Versus Other Ectodermal Tissues. Heat-map of the most differentially expressed genes in lacrimal gland versus other tissue types (top 50). Only genes that have FDR <0.01 in all pairwise comparisons and prioritized by average fold change either up or down regulated between lacrimal gland and each other tissue. The inset pie chart shows the total number of differentially expressed genes which are up, down or ambiguously differentially expressed.

We then sought to further investigate the genes that were expressed differentially in lacrimal gland versus other tissues. Fig 4 is a heat-map of the top 50 genes that are differentially expressed in lacrimal gland versus all other tissues in pair-wise comparisons. The inset pie-chart shows the number of lacrimal gland specific genes overall which were up, down or ambiguously (up in some pair wise comparisons, down in others, but always different) expressed in lacrimal gland versus other tissues. Almost all lacrimal gland-specific genes were consistently up- or down-regulated for each tissue comparison.

**Fig 5:**
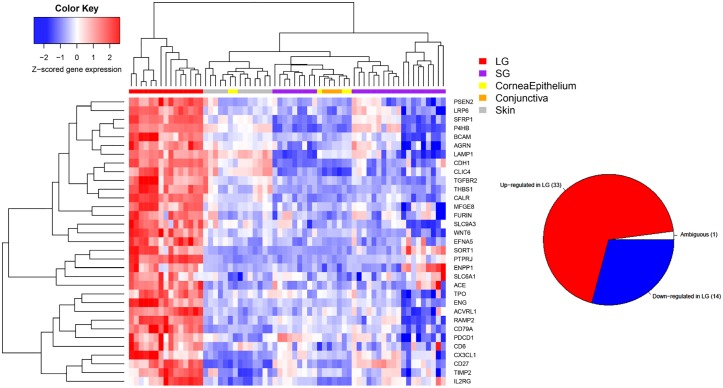
Heat-Map of Cell Surface Genes in Lacrimal Gland Versus Other Ectodermal Tissues. Heat-map of the up-regulated lacrimal gland-specific cell surface genes in lacrimal gland versus other tissue types. The inset pie chart shows the total number of cell surface genes which are up, down or ambiguously differentially expressed.

After defining the top 50 differentially expressed genes in lacrimal gland, we sought to specifically investigate the differentially expressed cell surface genes in an attempt to identify and define lacrimal-tissue specific surface markers. Fig 5 shows the cell surface genes that are lacrimal gland-specific and consistently up-regulated; the inset pie-chart shows the overall up, down and ambiguously differentially expressed cell surface genes in lacrimal gland-specific genes.

**Fig 6:**
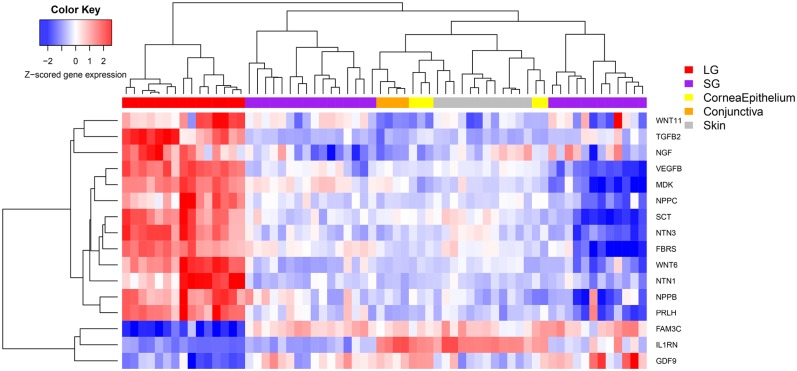
Heat-Map of Genes for Secreted Proteins Expressed in Lacrimal Gland Versus Other Ectodermal Tissues. Heat-map of differentially expressed genes that encode for secreted proteins in lacrimal gland versus other tissue types.

We were also interested in understanding the differential expression of genes that encode secreted proteins from lacrimal gland versus other tissue types and this data is presented in Fig 6.

**Fig 7:**
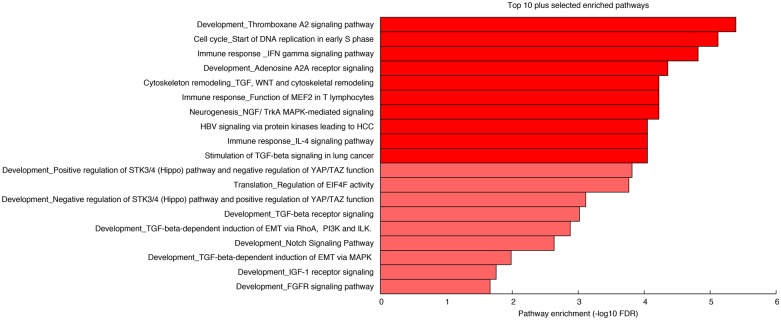
Differentially Regulated Signaling Pathways in Lacrimal Gland. Bar plot showing the top 10 differentially regulated developmental signaling pathways in lacrimal gland in addition to 9 (depicted in a lighter shade of red) pathways of potential interest in lacrimal gland development.

Once we had evaluated the differential gene expression of lacrimal gland specific, cell surface and secreted protein encoding genes, we sought to better understand which signaling pathways were differentially expressed in lacrimal gland. Fig 7 shows the enrichment significance (-log10 FDR) for the top 10 pathways (MetaCore’s^®^ pathway maps) differentially regulated in lacrimal gland in addition to 9 pathways which may be of interest in lacrimal gland development.

**Fig 8:**
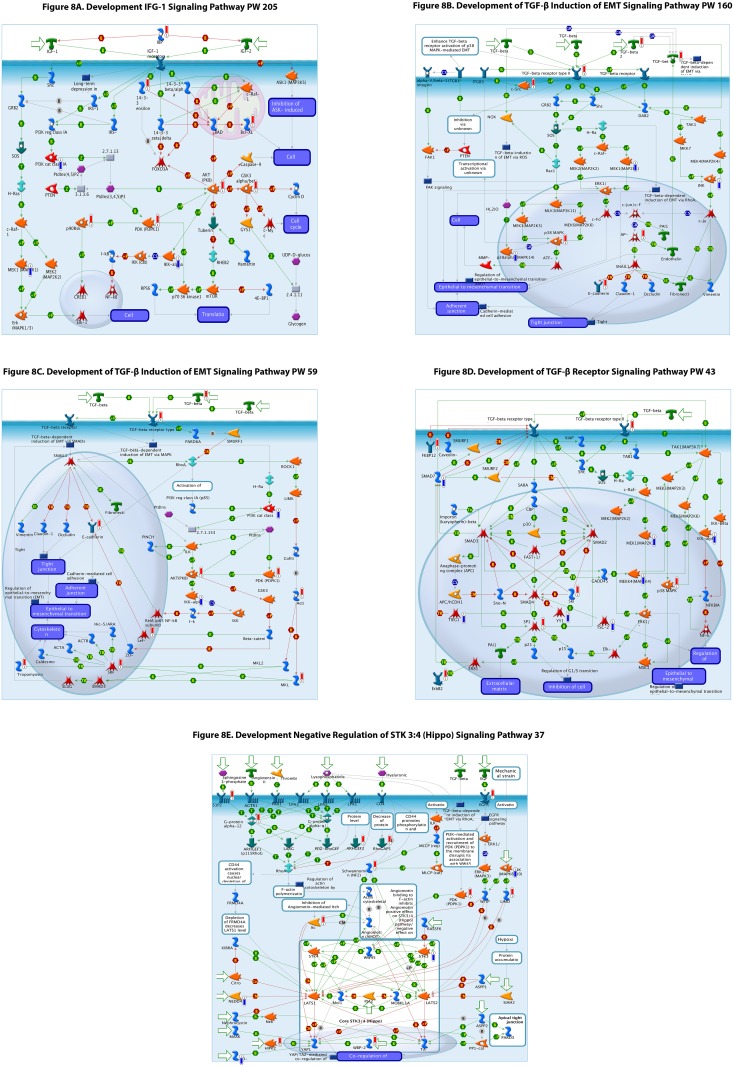
(A-E) Selected signaling pathway schematics demonstrating increase (red bar) or decrease (blue) bar and relative amplitude (height of bar) of differentially expression in each gene and their roles in the signaling pathway depicted. Green arrows indicate positive interactions or activation, red arrows indicate negative interactions or inhibition, while grey arrows indicate an unknown interaction. More details of symbol meanings can be found in the supplemental tables in the Metacore ^®^ quick reference guide.

To better understand these pathways we utilized the MetaCore ^®^ database to visualize the signaling pathways that were differentially regulated in lacrimal gland. Selected pathway schematics are shown and increases (red bar) and decreases (blue bar) in gene expression for each gene that is differentially expressed are depicted (Fig 8A–8E).

**Fig 9:**
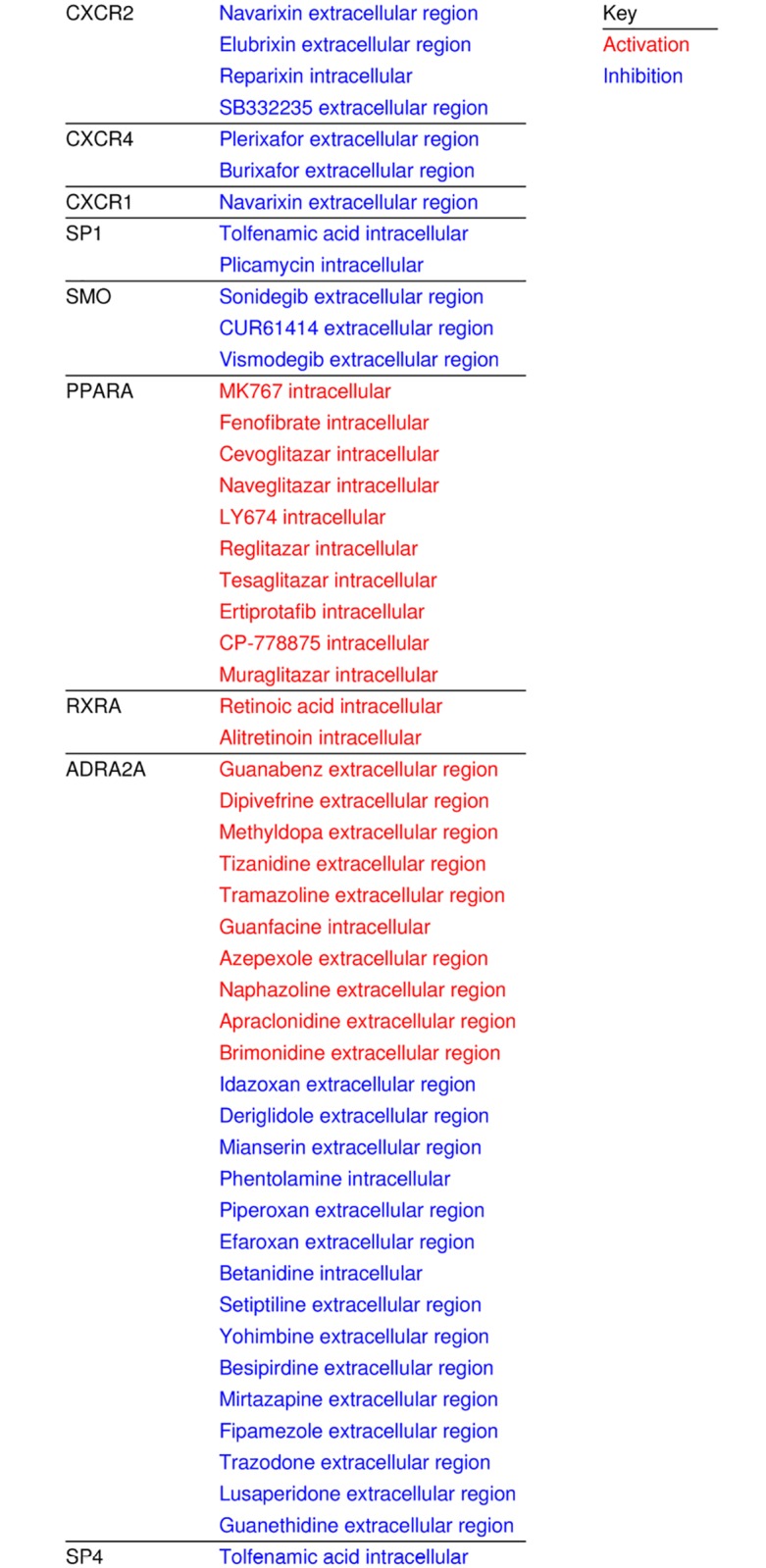
List of Drugs and Their Action Targeting Differentially Expressed Genes in Lacrimal Gland. Drugs categorized by differentially expressed targeted genes in lacrimal gland and their action (inhibition blue, activation red).

Finally we investigated the potential gene targets and their targeting drugs and molecules which are differentially expressed in lacrimal gland and these are shown in Fig 9.

## Discussion

This study demonstrates the “unique” nature of the human lacrimal gland when compared to other human surface ectoderm derived tissue types. There are a number of diseases which affect both the lacrimal gland and ocular surface (DES) and the lacrimal gland and salivary gland, such as Sjogren’s disease. There have been a number of studies demonstrating overlap in gene expression and cell types in multiple exocrine tissues [18]. However, modern medicine has moved towards a tailored approach to disease which takes into account patient specific factors in addition to organ and tissue specific factors to minimize side effects and enhance efficacy. Developing a better understanding of what genes are expressed in human lacrimal gland will provide a step towards the creation of tailored therapies for lacrimal gland diseases.

Fig 2 demonstrates the robust differences in gene expression between LG and various other tissues. This highlights the need for the development of a better understanding of lacrimal gland biology and gene expression. Some differences were noted on PCA between lacrimal gland tissues which clustered slightly differently. We attribute this to lab/batch effect as one group was from tissue from our group and the others are from GEO database obtained data on different microarray platforms, but these effects are small compared to the tissue differences that are clearly observed in the PCA plot. In addition, when evaluating clustering on the PCA plot, we did not note significant differences between the main lacrimal gland samples and accessory lacrimal gland samples from our laboratory indicating that these tissue types could be reasonably lumped together for analysis in this context. In other words, in this study we find that accessory lacrimal gland gene expression is more similar to main lacrimal gland than it is to the other tissue types evaluated, which is what might be expected. Fig 4 demonstrates the differential expression of the top developmentally important genes in lacrimal gland versus other tissue types. Of note is the strongly different gene expression between lacrimal gland and each tissue type. In addition, most differentially expressed genes were consistently either upregulated or downregulated in lacrimal gland, as opposed to a very small number of ambiguously differentially expressed genes which were up regulated in lacrimal gland compared with some tissues and downregulated compared with others. This may indicate a developmentally unique gene expression pattern that could be utilized for further study.

Development of phenotypic markers for lacrimal cell types requires an understanding of the differential gene expression of genes that code for cell surface proteins. Fig 5 shows these genes and the number of genes which were increased or decreased in lacrimal gland. Further studies doing immunofluorescence localization and protein based assays will be important to further evaluate these candidate genes.

Understanding the differential expression of genes encoding secreted proteins in lacrimal gland may enhance the study of tear proteomics and other important areas of study in ectodermal biology from a different vantage point. Fig 6 shows the genes which encode for secreted proteins and are differentially expressed in lacrimal gland.

The development of the lacrimal gland and branching organogenesis has been the topic of multiple studies of late [8, 11, 19]. These studies in addition to our own gene expression studies which have shown the presence of stem cell markers in adult lacrimal tissue are important to developing regenerative technologies for lacrimal gland atrophy, disease and hypofunction. Understanding the expression of the developmental pathways in adult lacrimal tissue could be instrumental in the development of regenerative medicine efforts for the lacrimal gland. Some significant efforts have already been made in these areas [2, 11]. Moreover, studies utilizing directed differentiation from iPS or ES cells to drive primitive cells to a lacrimal fate may require an understanding of the expression of genes in adult tissue [20].

Fig 7 shows the top differentially regulated signaling pathways in addition to 9 pathways which may be of interest in lacrimal gland development. Interestingly we found that TGF beta and Wnt signaling pathways were highly differentially regulated. In addition, we found multiple aspects of the FGF signaling pathway that were differentially regulated in lacrimal gland. This is consistent with recent studies on FGF signaling and lacrimal gland development [21–22].

We further analyzed these pathways through pathway schematic visualization using MetaCore ^®^ and selected pathways in Fig 8 are revealing. We note that there are significant differential expression changes in multiple levels of the TGF beta pathway, IGF-1 pathway, FGF pathways and hippo pathways which may be of interest for further study and analysis (S1–S6 Figs are included depicting other signaling pathways of interest) [8, 23,24].

Finally, gene expression analysis can be helpful in driving the development or repurposing of available therapeutics for diseases or studies targeted for specific cell types. Fig 9 shows differentially expressed genes which are targets of known molecules and drugs and their actions on those genes in lacrimal tissue. These drugs could be utilized in the development of future studies. The drugs and genes related to the targets of these drugs are relevant in a number of systemic and lacrimal gland diseases. CXCR2 in particular has been implicated as having importance in Sjogren’s syndrome [25]. Drugs targeting CXCR2, such as reparixin have been utilized clinically [26]. CXCR 4 has been seen as a potential marker of mucosa associated lymphoid tissue tumors in extragastric tissue [27]. Lymphoma is a common tumor in lacrimal gland [28]. Plerixafor has been utilized as an adjunctive therapy, to mobilize hematopoietic stem cells, in patients with lymphomas [29]. Repurposing drugs and deeper investigation of potentially available drugs and their targets may be important in the development of new treatment paradigms for lacrimal gland diseases.

In addition to better understanding of lacrimal gland diseases through evaluation of gene expression analysis, development of regenerative therapies could be enhanced by better data on the differences between lacrimal gland and other embryologically similar tissue types. Directed differentiation experiments [20,30], in which immature stem cells are differentiated into mature cell types using changes in gene expression depend on a detailed understanding of critical developmental factors. Several studies have demonstrated the ability of stem cells to be differentiated into ectodermal cell types, but none have been reported on directing differentiation into lacrimal epithelial cells [20]. We believe a strong understanding of the differential gene expression and signaling pathway differences between lacrimal gland and other ectodermal tissue types will be critical in the advancement of stem cell methodologies to create lacrimal epithelium *in vitro*. Moreover, limited information is available on what cell surface markers are present in lacrimal epithelium [7]. The data presented herein can serve as a basis for further targeted studies to define lacrimal epithelial and other cell phenotypic markers for future study.

Limitations of this study include a limited number of available samples in some tissue types, the need for comparison across multiple gene array types, and lack of data on splice variants. Strengths of this study include a robust data analysis utilizing data from multiple laboratory groups (limiting batch effects), an overall large sample size and large number of statistically significant gene differentials a number of which have corroborating evidence in other types of studies from the literature.

## Conclusion

We believe this is the first study to compare microarray based gene expression of normal human lacrimal gland to other embryologically related surface ectoderm derived human tissues. These data represent progress in the development of a more complete understanding of lacrimal gland specific gene expression, and may form the basis for future studies on drug development, disease and organ specific gene expression, developmental studies and regenerative medicine efforts.

## Supporting Information

S1 Fig Development FGFR Signaling Pathway PW 222. (EPS)

S2 Fig Development Notch Signaling Pathway PW 75. (EPS)

S3 Fig Translation Regulation ELF4F Activity Signaling Pthway PW 20. (EPS)

S4 Fig Development Negative Regulation of STK3:4 (Hippo) Signaling Pathway PW 12. (EPS)

S5 Fig Development FGF Family Signaling Pathway PW 376. (EPS)

S6 Fig Metacore Quick Reference Guide. (EPS)
